# Global warming intensifies the interference competition by a poleward-expanding invader on a native dragonfly species

**DOI:** 10.1098/rsos.230449

**Published:** 2023-11-22

**Authors:** Koki Nagano, Masayoshi K. Hiraiwa, Naoto Ishiwaka, Yugo Seko, Koya Hashimoto, Taizo Uchida, Francisco Sánchez-Bayo, Daisuke Hayasaka

**Affiliations:** ^1^ Graduate School of Agriculture, Kindai University, Nakamachi 3327-204, Nara, Nara 631-8505, Japan; ^2^ Faculty of Agriculture, Kindai University, Nakamachi 3327-204, Nara, Nara 631-8505, Japan; ^3^ National Institute for Environmental Studies (NIES), Onogawa 16-2, Tsukuba, Ibaraki 305-8506, Japan; ^4^ Faculty of Agriculture and Life Science, Hirosaki University, Bunkyo-cho 3, Hirosaki, Aomori 036-8561, Japan; ^5^ Faculty of Architecture and Civil Engineering, Kyushu Sangyo University, Higashi-ku, Matsukadai 2-3-1, Fukuoka, Fukuoka 813-8503, Japan; ^6^ School of Life and Environmental Sciences, The University of Sydney, Sydney, New South Wales 2006, Australia

**Keywords:** biological invasion, climate change, competitive displacement, ecological risk, foraging capacity/behaviour, interspecific interactions

## Abstract

Rapid climate warming has boosted biological invasions and the distribution or expansion polewards of many species: this can cause serious impacts on local ecosystems within the invaded areas. Subsequently, native species may be exposed to threats of both interspecific competition with invaders and temperature rises. However, effects of warming on interspecific interactions, especially competition between invader and native species remains unclear. To better understand the combined threats of biological invasions and warming, the effect of temperature on competitive interactions between two dragonfly species, the expanding *Trithemis aurora* from Southeast Asia and the Japanese native *Orthetrum albistylum speciosum* were assessed based on their foraging capacity. Although the stand-alone effect of temperature on foraging intake of the native dragonfly was not apparent, its intake significantly decreased with increasing temperatures when the invader *T. aurora* was present. Such reductions in foraging might lead to displacement of the native species through competition for food resources. This suggests that impacts of invader species against native species are expected to be more severe when interspecific competition is exacerbated by temperature rises.

## 1. Introduction

Global warming has been increasing at an accelerated rate [1] and seriously having an impact on various organisms and ecosystems [2–5]. Especially, the poleward expansion or migration of species that originated in low latitudes is a well-known consequence of the warming [6–8]. For example, temperature increases have facilitated the invasion of a dengue mosquito in northern Italy [9], expanded poleward the geographical distribution of dragonflies in the UK [10], and it is predicted that coral habitats will extend poleward over several hundred kilometres during this century [11]. However, biological invasions and expansions owing to warming occur not only horizontally, i.e. in latitude, but also vertically, in altitude [8,12], and may severely impact on biological interactions in the invaded regions [13,14].

In general, species have climatic adaptations to their native habitat ranges [15,16]. Thus, invader species that originally evolved in warm areas would be more adaptable to heat than native species in colder regions [17,18]. In this case, the invaders might have a competitive advantage over the native species when exposed to increasing temperatures. For example, indirect interspecific competition for resources (i.e. exploitation competition) has been implied between plants of lowland mountain areas that migrate in altitude and compete with native alpine plants as the temperature rises owing to warming, subsequently resulting in species displacement [19,20]. Previous studies on competition attributed to warming between invaders and native species have mostly focused on plant species [14,19–21], as factors affecting the competition are relatively easy to detect because, unlike animals, plants do not move and their habitat requirements are fewer. The main competition among plants would be for natural resources such as light, water and nutrients [22]. By contrast, animal competition has more complex mechanisms, including not only indirect competition for food resources (e.g. scramble for food) but also direct fighting or attack (interference competition) between invading and native species [23–28]. Therefore, animal invasions are likely to have more severe ecological impacts on local communities and ecosystems owing to the intensification of competition not just by exploitation of resources but also by interfering directly with native species. The influence of climate warming on these two types of competition between invaders and native species has not been assessed yet, despite awareness of the importance of such impacts.

**Figure 1.**
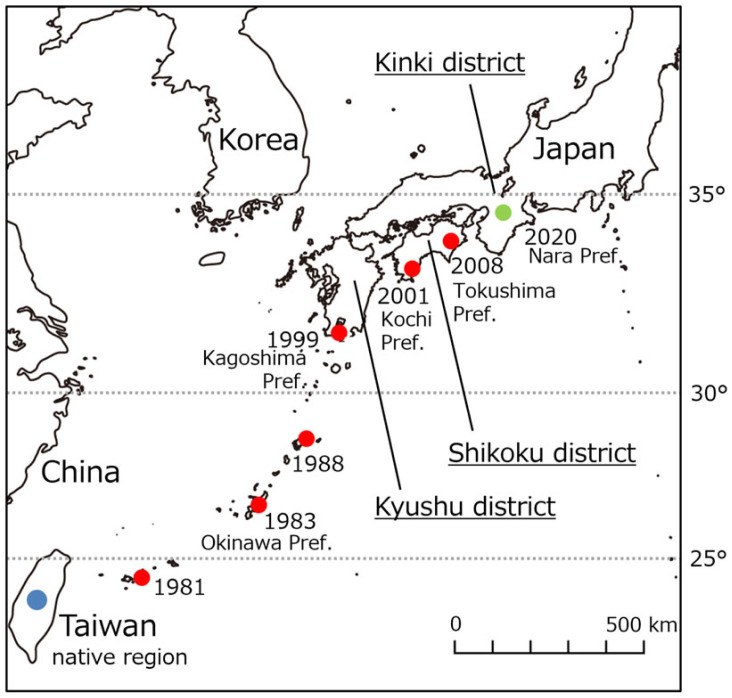
Tendency of the distribution expansion of the invasive *Trithemis aurora* in Japan. *Trithemis aurora*, native to Southeast Asia and Taiwan (subtropical to tropical climates), was first detected in subtropical Ishigaki Island (southwest Japan) in 1981 [34,35], and then collected in Okinawa Island in 1983 [34], Amami-Oshima Island in 1988 [33], and the southern Kyushu district in 1999 [34]. Its distribution had spread to the Shikoku district by the late 2000s [33,36]. Furthermore, distribution of *T. aurora* extended to the central Kinki district (Nara Prefecture) in 2020. Solid blue and red circles show native of *T. aurora* and its invaded regions, respectively. The solid green circle indicates the latest area of *T. aurora* detection (Nara Prefecture, central Kinki district).

Among animal invader species, dragonflies constitute a taxon known for their long-range movement [29,30]. In fact, some species like the crimson marsh glider (*Trithemis aurora* Burmeister) have expanded poleward owing to recent warming [31,32]. *Trithemis aurora*, which is native to Southeast Asia and Taiwan [33], was first detected in Yaeyama and Okinawa Islands of southwest Japan around the 1980s, and then moved towards temperate latitudes in the Shikoku district by the late 2000s. Furthermore, distribution of *T. aurora* extended to the central Kinki district (Nara Prefecture) in 2020 (figure 1). By contrast, the Japanese white-tailed skimmer (*Orthetrum albistylum speciosum* Selys), which shares similar ecological characteristics with *T. aurora,* at least at the nymphal stage, is the most common and abundant native dragonfly in Japan. Both dragonfly species ordinarily occur among the vegetation and surface layer of the muddy soil of lentic environments, including rice paddies in Japan [37,38] and thus, can frequently be collected at the same sites. It is also known that dragonfly nymphs of these two species have a sharp dynamic hook at the tip of the lower lip and they prefer similar prey [38,39]. Dragonfly nymphs are important predators in aquatic ecosystems and often become top predators in habitats without fishes [40,41]. Furthermore, both dragonflies belong to the same family (Libellulidae), have similar body size and growth period, and this can cause interspecific competition, especially during their nymphal stage [42,43]. Competition for resources among dragonfly nymphs can indirectly affect their foraging and mortality [44,45], and the possibility that they can attack and prey on each other cannot be denied [46]. Naturally, their foraging intake may also change as a result of being attacked. If the interspecific competition between both species is severe owing to these two aspects of competition (i.e. exploitation and interference), it is envisaged that the effect of warming on *T. aurora* may aggravate the already negative impact the invader has on *O. albistylum speciosum* populations and lead to competitive displacement of the native species.

In this paper, we intend to answer the following two questions: (i) does foraging intake of the invader *T. aurora* and native *O. albistylum speciosum* dragonfly nymphs change in response to increasing temperature? and (ii) is the foraging behaviour of the two species modified owing to interspecific competition when facing a resource competitor?

## 2. Material and methods

### 2.1. Information on dragonfly species tested and their characteristics

*Orthetrum albistylum speciosum* is a native species in central Kinki district (Nara Prefecture), a region that *T. aurora* has only recently invaded. These species were selected to predict threats of biological invasions in the near future.

Life-history characteristics of the two dragonflies species are as follows: *O. albistylum speciosum* is known to be multivoltine, and *T. aurora* can be either multivoltine or univoltine. In addition, although there is no evidence from the literature that either species overwinter as eggs, it is known that their nymphal stages overwinter [47].

### 2.2. Collection and breeding of test species

On 5 July 2021, adult females of *O. albistylum speciosum* were sampled from the campus of Kindai University, Nara (34°40′22″ N, 135°43′47″ E). On 12 July 2021, adult females of non-native *T. aurora* were sampled from Kaiyo town, Tokushima (east Shikoku) (33°37′07″ N, 134°22′27″ E), which is the northernmost point of its established range in Japan. After collection, the tip of the abdomen was inserted in a centrifuge tube filled with dechlorinated tap water (rearing water) to induce the production of eggs.

Eggs of both species were reared separately in plastic cups (66 mm diameter × 36 mm depth) containing 40 ml of rearing water, and the nymphs that emerged from the eggs were used in this experiment. Eggs and nymphs were kept in an incubator (LH-30-8CT, Nippon Medical & Chemical Instruments Co, Ltd., Osaka, Japan) that controlled the following three temperatures tested in separate treatments: 27, 29 and 31°C. The lowest temperature (27°C) represents the control, and was chosen as the 20-year average temperature of the warmest month during the activity period of *O. albistylum speciosum* nymphs at the collection site (https://www.data.jma.go.jp/obd/stats/etrn/index.php). The other two treatments follow the global warming scenarios set by the Intergovernmental Panel on Climate Change AR6 [1], which indicate increases of +2°C (SSP1-2.6) and +4°C (SSP5-8.5) with respect to the control. By contrast, the warmest monthly average temperature during the activity period of *T. aurora* in its native region (Taiwan) was about 31°C during the past decade (https://www.cwb.gov.tw/V8/E/C/Statistics/monthlydata.html), which is the same as the highest treatment in our experiment. Also, an incubator photoperiod was set at 14 L : 10 D hours with reference to the sunlight hours of the warmest month in the collection site of *O. albistylum speciosum* nymphs, and this setting was based on data from the National Astronomical Observatory of Japan (https://eco.mtk.nao.ac.jp/koyomi/dni/2021/s3008.html). Nymphs of both species hatched within 6–10 days after eggs were collected, and then reared individually in plastic cups (66 mm diameter × 36 mm depth). Nymphs of both species were fed nauplius larvae of *Artemia salina* L. (tetra brine shrimp eggs, Spectrum Brands Japan, Inc, Kanagawa, Japan) once every second day.

### 2.3. Foraging tests

To verify effects of an invading competitor on the foraging behaviour of native species, predation experiments of *T. aurora* and *O. albistylum speciosum* nymphs were performed using the method of De Block *et al*. [48] and Inouye [49] with some modifications. To remove the effect of time and/or photoperiod, all foraging tests were conducted only during the daytime when the light in the incubator was turned on (i.e. 10.00 to 18.00). Foraging intakes of *A. salina* nauplius larvae were firstly tested on each species under the situation without a competitor (stand-alone test). Test procedures are as follows: (i) for each temperature treatment and species, 9–10 replicates were used, each replicate consisting of a plastic cup (see above) that contained one individual; (ii) nymphs about two-months old after hatching (about 4–8 mm in size) were selected randomly and transferred to plastic cups containing 40 ml of rearing water; (iii) after 1 h, thirty *A. salina* larvae per cup were introduced, and then the experiment was started; and (iv) the number of *A. salina* remaining 2 h after the experiments was counted to estimate the maximum foraging intake of each species.

To examine changes in the foraging intake of each species attributed to the presence/absence of a competitor, an experiment was conducted under the condition that both species were present in a cup (cohabitation test) with reference to additive designs which can detect a significant effect of interspecific competition on the performance of a focal species [49], and the results of this test were compared to the results of the stand-alone test. Procedures were almost the same as in the stand-alone experiment, the main difference being that each cup contained two nymphs randomly paired, one of each species. To remove effects of differences among individuals, this experiment used the same individuals as those used in the stand-alone tests. In the cohabitation experiment, however, 60 *A. salina* larvae were introduced into the plastic cups to account for the two nymphs present. This design enabled us to discern the effects of interference competition from the exploitation competition that would result from a decrease in available food.

To determine the effect of temperature on the competitive interference between dragonflies, we counted the number of attacks among the two species. Both foraging intake and attacks were observed during 2 h in each experiment. Foraging intake was defined as the number of *A. salina* eaten per nymph. An attack was defined as making contact with the other species and having the touched individual turned its head away from the attacker. When one competitor preyed on the other, data on the foraging intakes were excluded from analyses.

To understand the effect of body size on the competition, the body length as an indicator of body sizes (length from the head without antenna to the abdomen including caudal appendages) of all individuals tested were measured before the experiments.

### 2.4. Statistical analysis

To test effects of temperature increases and the presence/absence of a competitor on foraging intakes of each species, we used generalized linear mixed models (GLMMs; with Poisson errors and log link), where the foraging intake of *O. albistylum speciosum* or *T. aurora* nymphs was the response variable in the stand-alone and cohabitation tests. The explanatory variables were as follows: temperature, presence/absence (p/a) of a competitor, body size and their interactions (temperature × competitor, body size × competitor). Also, individual differences in each dragonfly nymph tested were fitted as random effects. Replicates in which predation from a competitor occurred during the monitoring period were excluded from the analysis. Furthermore, relationships between the number of attacks from a competitor during the experiments and temperatures were analysed using generalized linear models (GLMs; with Poisson errors and log link), with the number of attacks from each species as the response variable and temperatures as the explanatory variable in the cohabitation test. All statistical analyses were conducted using R v. 4.1.3 [50]. GLMs and GLMMs were conducted using the package ‘glmmTMB’ [51].

## 3. Results

**Figure 2.**
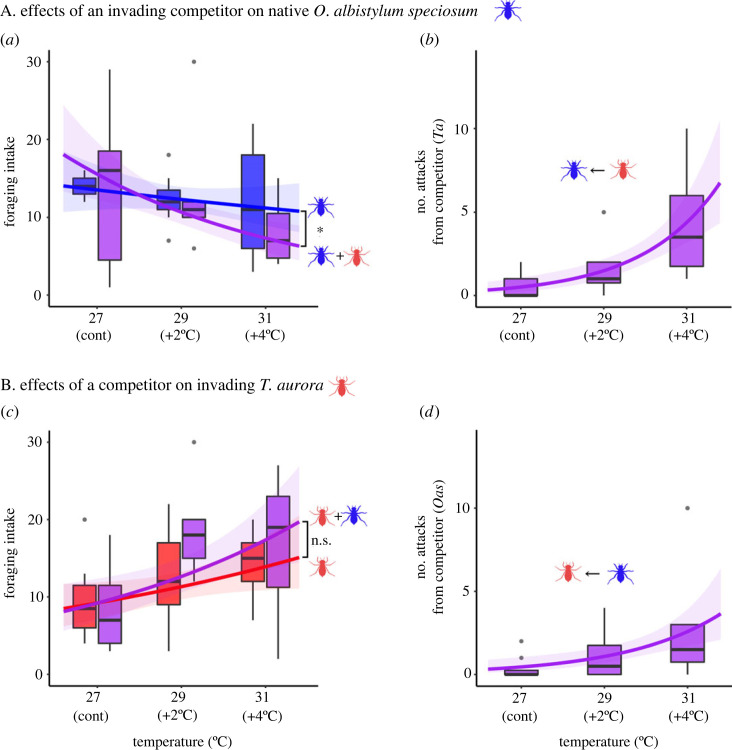
A. Effects of the invading competitor *T. aurora* (*Ta*) on native *O. albistylum speciosum* (*Oas*)*.* (*a*) Effect of temperature and presence of the invader on the foraging intake of the native species under two experimental conditions: stand-alone (blue), and cohabitation (purple); (*b*) number of attacks of *T. aurora* on *O. albistylum speciosum*. B. Effects of a native competitor on invading *T. aurora*. (*c*) Effects of temperature and presence or absence of *Oas* on the foraging intake of *Ta* under the two experimental conditions: stand-alone (red) and cohabitation (purple); (*d*) number of attacks of *O. albistylum speciosum* on *T. aurora*. Fitted curves and bands for 95% confidence interval with GLMMs and GLMs. In (*a*) and (*c*) the asterisks indicate that the interaction between temperature and the presence or absence of competing species is significant at *p* < 0.05 and n.s. indicates no significant difference.

Although the foraging intake of *O. albistylum speciosum* nymphs did not differ among temperatures in the stand-alone test, the intake of this species significantly decreased with increasing temperatures when *T. aurora* was present (temperature: *z* = −1.027, *p* = 0.305; temperature × p/a of a competitor: *z* = −2.318, *p* = 0.021; figure 2*a* and table 1). The number of attacks by *T. aurora* on the native species increased significantly with increasing temperatures (*z* = 4.460, *p* < 0.001; figure 2*b*). By contrast, the foraging intake of *T. aurora* significantly increased with increasing temperature irrespective of the presence of a competitor or not (temperature: *z* = 2.079, *p* = 0.038; temperature × p/a of a competitor: *z* = 1.078, *p* = 0.281; figure 2*c* and table 2). Similarly, the number of attacks from *O. albistylum speciosum* on the invader increased significantly with increasing temperatures (*z* = 3.487, *p* < 0.001; figure 2*d*).

**Table 1.** Effects of temperature, presence/absence (p/a) of a competitor and body size on foraging intake of *Orthetrum albistylum speciosum* nymph using GLMMs (*p < 0.05, ^†^*p* < 0.1).

variables	estimate	s.e.	*z*-value	*p*-value
temperature	−0.047	0.046	−1.027	0.305
p/a of a competitor	2.945	1.574	1.871	0.061^†^
body size	−0.009	0.055	−0.156	0.876
temperature × p/a of a competitor	−0.141	0.061	−2.318	0.021*
body size × p/a of a competitor	0.162	0.069	2.352	0.019*

**Table 2.** Effects of temperature, presence/absence (p/a) of a competitor and body size on foraging intakes of *Trithemis aurora* nymph using GLMMs (*p < 0.05).

variables	estimate	s.e.	*z*-value	*p*-value
temperature	0.102	0.049	2.079	0.038*
p/a of a competitor	−1.499	1.614	−0.929	0.353
body size	−0.089	0.077	−1.156	0.248
temperature × p/a of a competitor	0.056	0.052	1.078	0.281
body size × p/a of a competitor	−0.003	0.074	−0.043	0.966

**Figure 3.**
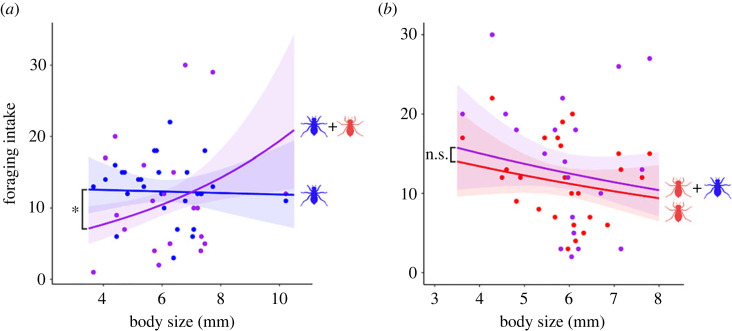
(*a*) Effect of the body size and the presence or the absence of *T. aurora* on the foraging intake of *O. albistylum speciosum* under the two experimental conditions: stand-alone (blue) and cohabitation (purple). (*b*) Effect of the body size and presence of *O. albistylum speciosum* on the foraging intake of *T. aurora* under the same two conditions: stand-alone (red) and cohabitation (purple). Fitted curves and bands for 95% confidence interval with GLMMs. The asterisks indicate that the interaction between temperature and the presence or the absence of competing species is significant at *p* < 0.05 and n.s. indicates no significant difference.

The foraging intake of *O. albistylum speciosum* did not change regardless of its body size in the stand-alone test, whereas the intake significantly decreased in the smaller individuals when tested under the cohabitation condition (body size: *z* = −0.156, *p* = 0.876; body size × p/a of a competitor: *z* = 2.352, *p* = 0.019; figure 3*a* and table 1). However, *T. aurora* had similar foraging intake irrespective of its body size and the presence or absence of the native competitor (body size: *z* = −1.156, *p* = 0.248; body size × p/a of a competitor: *z* = −0.043, *p* = 0.966; figure 3*b* and table 2).

Furthermore, *O. albistylum speciosum* nymphs were preyed upon by *T. aurora* in all temperatures tested (three individuals at 27/29°C, one at 31°C).

## 4. Discussion

Our study provides a new perspective about the threats that biological invasive species pose to native species via interference competition along with warming. These two components have rarely been discussed, and we have shown that ecological impacts can be more severe with rising temperatures. As we expected, the foraging behaviour of the native dragonfly species (*O. albistylum speciosum*) was negatively affected and resulted in a decrease in foraging intake through encounters with the invading species (*T. aurora*). Although the foraging intake of *O. albistylum speciosum* in situations without a competitor was similar irrespective of the temperature, such intake significantly decreased with increasing temperatures when the invader *T. aurora* was present (figure 2*a*). This indicates that adverse impacts of invaders on native species via food acquisition may become more severe with current and future warming of the planet.

The number of attacks by *T. aurora* on the native species clearly increased with temperature (figure 2*b*). This phenomenon may be associated with better performance of *T. aurora* under warmer conditions because this species inhabits subtropical zones and is naturally adapted to such conditions. Given that the resource acquisition rate is related to the survival rate of a species [52], our results support previous studies showing that survival rates of invaders from warmer regions can be higher when temperature rises [17]. Since the attacking behaviour of *T. aurora* is an important factor which directly diminishes the feeding ability of *O. albistylum speciosum*, this native species will be exposed to further adverse effects in terms of food acquisition when the temperature rises in the presence of the invader competitor, and this combined effect will inevitably lead to a decrease of its population. In addition, while the stand-alone experiment demonstrated that the foraging intake of *O. albistylum speciosum* nymphs remains unaltered irrespective of its body size, the smaller nymphs experienced higher foraging loses when confronted with the invader *T. aurora* (figure 3). This suggests that the behaviour of *T. aurora* was more effective against the smaller nymphs of *O. albistylum speciosum*, which were outcompeted by the invader. In response to these findings, we tested the relationship between the body size of *O. albistylum speciosum* and the body size difference of *O. albistylum speciosum* and *T. aurora*. The result showed a significant relationship between the two factors (*r* = −0.81, *t*_18_= −5.86, *p* < 0.001; Pearson's correlation test; electronic supplementary material, figure S1). In other words, *O. albistylum speciosum* individuals, which are relatively smaller than *T. aurora*, are exposed to a stronger impact in relation to foraging ability. Conversely, the larger *O. albistylum speciosum* individuals will have a smaller impact. This suggests a size-mediated priority effect [53,54]. By contrast, although *O. albistylum speciosum* clearly increased its attacks towards the invader under situations of higher temperatures (figure 2*d*), the foraging intake of *T. aurora* increased regardless of the existence of a competitor (figure 2*c*), thus suggesting the attacking behaviour of the native did not impact on the foraging ability of the invader. In other words, the foraging intake of *O. albistylum speciosum* is negatively affected by *T. aurora* invasion under higher temperature conditions, but not vice versa.

Given that both types of competition, interference and exploitation probably occur among dragonfly nymphs in resource-limited environments [45], the increasing fitness of *T. aurora* under warm conditions suggests this species is a real threat towards native dragonflies owing to global warming. Indeed, expected temperature rises may intensify the interspecific competition among dragonflies owing to both exploitation and interference competition. Moreover, the unilateral predation of *T. aurora* upon *O. albistylum speciosum* can be described as asymmetrical intraguild predation, which is one of the mechanisms causing species displacement [55], since this predation was observed in all temperatures in cohabitation tests despite feeding the nymphs saturated amounts of food.

Our primary objective was to quantify the effects of invasive competitors attributed to warming on the food acquisition performance of native species, not to compare the relative strength of its inter- and intraspecific competitive effects. The observed asymmetric interspecific competition between *T. aurora* and *O. albistylum speciosum* under increasing temperatures may result in *T. aurora*'s competitive advantage, which could eventually lead to the displacement of the native *O. albistylum speciosum* in Japan. It should be noted that the effects of intraspecific competition are not considered in this experiment. To properly predict the outcomes of competitive coexistence (or displacement), both intra- and interspecific interaction parameters are needed. Moreover, in the real world, *O. albistylum speciosum* would compete not only with *T. aurora*, but also with other dragonfly species and also be exposed to antagonistic interactions with top predators such as fishes [54]. The presence of such ‘third species' may modify the interaction between *T. aurora* and *O. albistylum speciosum*, which makes it more difficult to predict the outcomes of *T. aurora* invasion. Thus, for properly extrapolating our experimental results to more complex field environments and predicting more accurately the ecological threats of *T. aurora* invasion we need to consider these perspectives comprehensively in future studies.

## 5. Conclusion

Our results support the hypothesis of the intensification of interference competition between invading and native species with increasing temperatures. This implies that impacts of biological invasions on native species through direct attacks might be exacerbated by global warming. Consequently, the impact of invader species from warmer regions on native species may be more severe than previous studies suggested because that competition for resources intensifies as the ambient temperate rises [19,20]. Our study proved this is the case for the two dragonfly species considered here. Thus, the possibility of a species displacement by a poleward-expanding invader through the two types of competitions (exploitation and interference) is real. However, our experiments were conducted within plastic cups containing only rearing water without any substrates (e.g. plants, mud and rock) that would allow the two dragonfly nymphs to hide. For instance, nymphs of *Aeshna viridis* Eversmann tend to avoid the intraguild predation by using vegetation as a refuge [56]. As mentioned before, *O. albistylum speciosum* nymphs often inhabit the vegetation or surface layer of the muddy soil in lentic environments [38], implying that they may avoid attacks from *T. aurora* by hiding. In addition, because in our study the two dragonfly nymphs tested were reared in the laboratory, the amount of food provided to these nymphs might have not been sufficient. Therefore, it is possible that the feeding frequency would have affected the competitive interactions, resulting in a different outcome to what occurs in the real world. To validate this prediction, it is important to assess not only interspecific competition between the two dragonfly nymphs but also competition with other species under experimental test systems that mimic the field environment (e.g. mesocosms).

How the impact posed by biological invasions to ecosystems change with warming is an important global issue, but it is very difficult to predict whether the impact will be severe or mild owing to the complexity of its process and mechanisms (e.g. [57]). On the other hand, our finding that 'threats by poleward-expanding species that invade from warmer regions become severe with warming' would be applied to other cases of biological invasions attributed to global warming. This is because poleward-expanding invaders originally inhabit warmer regions and thus, a further warming is expected to bring the invaded area closer to the temperature of their origin.

Recently, poleward-expanding invaders have been reported in various taxonomic groups and the importance of predicting their impacts is growing [6–12,17,18,58,59]. Our viewpoint should be applied to predict impacts of new invaders attributed to warming regardless of taxonomic groups. In summary, threats of biological invasions associated with warming are expected to be more severe than the effects of biological invasions alone owing to unbalanced interspecific competition through both exploitation and interference.

## Data Availability

We have added the URL on the climatic data in the sampling site of both dragonfly species (*Trithemis aurora* and *Orthetrum albistylum speciosum*) (see §2.2 *Collection and* *breeding of test species*). Supplementary material is available online [60].

## References

[1] IPCC. 2021 Climate change 2021: the physical science basis. In Contribution of working group I to the sixth assessment report of the Intergovernmental Panel on Climate Change (eds V Masson-Delmotte et al.). Cambridge, UK: Cambridge University Press. (https://www.ipcc.ch/report/ar6/wg1/#FullReport)

[2] Mann ME, Bradley RS, Hughes MK. 1999 Northern hemisphere temperatures during the past millennium: inferences, uncertainties, and limitations. Geophys. Res. Lett. **26**, 759-762. (10.1029/1999GL900070)

[3] Hughes L. 2000 Biological consequences of global warming: is the signal already apparent? Trends Ecol. Evol. **15**, 56-61. (10.1016/s0169-5347(99)01764-4) 10652556

[4] Walther G-R, Post E, Convey P, Menzel A, Parmesan C, Beebee TJC, Fromentin J-M, Hoegh-Guldberg O, Bairlein F. 2002 Ecological responses to recent climate change. Nature **416**, 389-395. (10.1038/416389a) 11919621

[5] Kudo G, Cooper EJ. 2019 When spring ephemerals fail to meet pollinators: mechanism of phenological mismatch and its impact on plant reproduction. Proc. R. Soc. B **286**, 20190573. (10.1098/rspb.2019.0573) PMC6571468 31185863

[6] Parmesan C et al. 1999 Poleward shifts in geographical ranges of butterfly species associated with regional warming. Nature **399**, 579-583. (10.1038/21181)

[7] Chen I-C, Hill JK, Ohlemüller R, Roy DB, Thomas CD. 2011 Rapid range shifts of species associated with high levels of climate warming. Science **333**, 1024-1026. (10.1126/science.1206432) 21852500

[8] Vaissi S. 2022 Response of Iranian lizards to future climate change by poleward expansion, southern contraction, and elevation shifts. Sci. Rep. **12**, 2348. (10.1038/s41598-022-06330-4) 35149739 PMC8837782

[9] Roiz D, Neteler M, Castellani C, Arnoldi D, Rizzoli A. 2011 Climatic factors driving invasion of the tiger mosquito (*Aedes albopictus*) into new areas of Trentino, northern Italy. PLoS ONE **6**, e14800. (10.1371/journal.pone.0014800) 21525991 PMC3078124

[10] Hickling R, Roy DB, Hill JK, Thomas CD. 2005 A northward shift of range margins in British Odonata. Global Change Biol. **11**, 502-506. (10.1111/j.1365-2486.2005.00904.x)

[11] Yara Y, Vogt M, Fujii M, Yamano H, Hauri C, Steinacher M, Gruber N, Yamanaka Y. 2012 Ocean acidification limits temperature-induced poleward expansion of coral habitats around Japan. Biogeosciences **9**, 4955-4968. (10.5194/bg-9-4955-2012)

[12] Gilgado JD, Rusterholz H, Baur B. 2022 Millipedes step up: species extend their upper elevational limit in the Alps in response to climate warming. Insect Conserv. Divers. **15**, 61-72. (10.1111/icad.12535)

[13] Tylianakis JM, Didham RK, Bascompte J, Wardle DA. 2008 Global change and species interactions in terrestrial ecosystems. Ecol. Lett. **11**, 1351-1363. (10.1111/j.1461-0248.2008.01250.x) 19062363

[14] Alexander JM, Diez JM, Levine JM. 2015 Novel competitors shape species' responses to climate change. Nature **525**, 515-518. (10.1038/nature14952) 26374998

[15] Bujan J, Ollier S, Villalta I, Devers S, Cerdá X, Amor F, Dahbi A, Bertelsmeier C, Boulay R. 2022 Can thermoregulatory traits and evolutionary history predict climatic niches of thermal specialists? Divers. Distrib. **28**, 1081-1092. (10.1111/ddi.13511)

[16] Guisan A, Petitpierre B, Broennimann O, Daehler C, Kueffer C. 2014 Unifying niche shift studies: insights from biological invasions. Trends Ecol. Evol. **29**, 260-269. (10.1016/j.tree.2014.02.009) 24656621

[17] Ohba S, Fukui M, Terazono Y, Takada S. 2020 Effects of temperature on life histories of three endangered Japanese diving beetle species. Entomol. Exp. Appl. **168**, 808-816. (10.1111/eea.12987)

[18] Gottfried M et al. 2012 Continent-wide response of mountain vegetation to climate change. Nature Clim. Change **2**, 111-115. (10.1038/nclimate1329)

[19] Pajunen AM, Oksanen J, Virtanen R. 2011 Impact of shrub canopies on understorey vegetation in western Eurasian tundra. J. Veg. Sci. **22**, 837-846. (10.1111/j.1654-1103.2011.01285.x)

[20] Mod HK, Luoto M. 2016 Arctic shrubification mediates the impacts of warming climate on changes to tundra vegetation. Environ. Res. Lett. **11**, 124028. (10.1088/1748-9326/11/12/124028)

[21] Okano K, Bret-Harte MS. 2015 Warming and neighbor removal affect white spruce seedling growth differently above and below treeline. SpringerPlus **4**, 79. (10.1186/s40064-015-0833-x) 25729635 PMC4339320

[22] Fernandez M, Malagoli P, Gallet C, Fernandez C, Vernay A, Améglio T, Balandier P. 2021 Investigating the role of root exudates in the interaction between oak seedlings and purple moor grass in temperate forest. For. Ecol. Manag. **491**, 119175. (10.1016/j.foreco.2021.119175)

[23] Davey AJH, Doncaster CP, Jones OD. 2009 Distinguishing between interference and exploitation competition for shelter in a mobile fish population. Environ. Model. Assess. **14**, 555-562. (10.1007/s10666-008-9171-5)

[24] Turra A, Denadai MR. 2004 Interference and exploitation components in interspecific competition between sympatric intertidal hermit crabs. J. Exp. Mar. Biol. Ecol. **310**, 183-193. (10.1016/j.jembe.2004.04.008)

[25] Reitz SR, Trumble JT. 2002 Competitive displacement among insects and arachnids. Annu. Rev. Entomol. **47**, 435-465. (10.1146/annurev.ento.47.091201.145227) 11729081

[26] Ode PJ, Vyas DK, Harvey JA. 2022 Extrinsic inter- and intraspecific competition in parasitoid wasps. Annu. Rev. Entomol. **67**, 305-328. (10.1146/annurev-ento-071421-073524) 34614367

[27] Bhuyain MMH, Lim UT. 2019 Interference and exploitation competition between *Frankliniella occidentalis* and *F. intonsa* (Thysanoptera: Thripidae) in laboratory assays. Fla. Entomol. **102**, 322. (10.1653/024.102.0206)

[28] DeLong JP, Vasseur DA. 2011 Mutual interference is common and mostly intermediate in magnitude. BMC Ecol. **11**, 1. (10.1186/1472-6785-11-1) 21211032 PMC3024213

[29] Stoks R, Córdoba-Aguilar A. 2012 Evolutionary ecology of Odonata: a complex life cycle perspective. Annu. Rev. Entomol. **57**, 249-265. (10.1146/annurev-ento-120710-100557) 21910638

[30] Khelifa R. 2019 Sensitivity of biodiversity indices to life history stage, habitat type and landscape in Odonata community. Biol. Conserv. **237**, 63-69. (10.1016/j.biocon.2019.06.010)

[31] Li F, Kwon Y-S, Bae M-J, Chung N, Kwon T-S, Park Y-S. 2014 Potential impacts of global warming on the diversity and distribution of stream insects in South Korea: stream biodiversity and global warming. Conserv. Biol. **28**, 498-508. (10.1111/cobi.12219) 24372690

[32] Kurashina H, Matsuki K, Horita M, Kano K, Hasegawa M. 2007 The dragonflies and damselflies of Oita prefecture. Beppu, Oita, Japan: Kyushu Research Group of Odonatology (in Japanese).

[33] Toyosaki I, Yamada K, Ôhara K. 2009 Records of *Trithemis aurora* (Odonata, Libellulidae) in Tokushima Prefecture, Shikoku, Japan. Bull. Tokushima Pref. Mus. **19**, 39-44.

[34] Watanabe K, Yakita R, Kohama T, Ozono A. 2007 The dragonflies of Okinawa. Chiyoda-ku, Tokyo, Japan: Ikadasha (in Japanese).

[35] Inoue K, Tani K. 2010 All about red dragonflies. Chuo-ku, Osaka, Japan: TOMBOW Publishing (in Japanese).

[36] Sugimura M, Kosaka K, Yoshida K, Ohama S. 2008 The dragonflies of Chugoku & Shikoku. Chiyoda-ku. Tokyo, Japan: Ikadasha (in Japanese).

[37] Kadoya T, Suda S, Washitani I. 2009 Dragonfly crisis in Japan: a likely consequence of recent agricultural habitat degradation. Biol. Conserv. **142**, 1899-1905. (10.1016/j.biocon.2009.02.033)

[38] Sugimura M, Ishida S, Kojima K, Ishida K, Aoki T. 1999 Dragonflies of the Japanese archipelago in color. Sapporo, Hokkaido, Japan: Hokkaido University Press (in Japanese).

[39] Inoue K, Tani K. 2017 All about dragonflies: worldwide revised edition. Chuo-ku, Osaka, Japan: TOMBOW Publishing (in Japanese).

[40] Nakanishi K, Yokomizo H, Hayashi TI. 2021 Population model analyses of the combined effects of insecticide use and habitat degradation on the past sharp declines of the dragonfly *Sympetrum frequens*. Sci. Total Environ. **787**, 147526. (10.1016/j.scitotenv.2021.147526) 34000531

[41] McPeek MA. 1990 Determination of species composition in the *Enallagma* damselfly assemblages of permanent lakes. Ecology **71**, 83-98. (10.2307/1940249)

[42] Ishida S, Ishida K, Kojima K, Sugimura M. 1988 Illustrated guide for identification of the Japanese odonata. Hiratsuka, Kanagawa, Japan: Tokai University Press (in Japanese).

[43] Tsubaki Y, Ubutaka H, Ueda T, Higashi K. 2007 Dragonflies: behavior and ecology of odonata. Shibuya-ku, Tokyo, Japan: Kaiyusha (in Japanese).

[44] Crowley PH, Dillon PM, Johnson DM, Watson CN. 1987 Intraspecific interference among larvae in a semivoltine dragonfly population. Oecologia **71**, 447-456. (10.1007/BF00378720) 28312994

[45] McPeek MA. 1998 The consequences of changing the top predator in a food web: a comparative experimental approach. Ecol. Monogr. **68**, 1-23. (10.1890/0012-9615(1998)068[0001:TCOCTT]2.0.CO;2)

[46] Worthen WB, Horacek HJ. 2015 The distribution of dragonfly larvae in a south Carolina stream: relationships with sediment type, body size, and the presence of other larvae. J. Insect Sci. **15**, 31. (10.1093/jisesa/iev013) 25843584 PMC4535471

[47] Ozono A, Kawashima I, Fytahashi R. 2012 Dragonflies of Japan. Shibuya-ku, Tokyo, Japan: Bun-ichi Co., Ltd. (in Japanese).

[48] De Block M, Pauwels K, Van Den Broeck M, De Meester L, Stoks R. 2013 Local genetic adaptation generates latitude-specific effects of warming on predator-prey interactions. Glob. Change Biol. **19**, 689-696. (10.1111/gcb.12089) 23504827

[49] Inouye BD. 2001 Response surface experimental designs for investigating interspecific competition. Ecology **82**, 2696-2706. (10.1890/0012-9658(2001)082[2696:RSEDFI]2.0.CO;2)

[50] R Core Team. 2022 R: a language and environment for statistical computing. Vienna, Austria: R Foundation for Statistical Computing. See https://www.r-project.org/.

[51] Brooks ME, Kristensen K, Van Benthem KJ, Magnusson A, Berg CW, Nielsen A, Skaug HJ, Mächler M, Bolker BM. 2017 glmmTMB balances speed and flexibility among packages for zero-inflated generalized linear mixed modeling. The R J. **9**, 378. (10.32614/RJ-2017-066)

[52] Petren K, Case TJ. 1996 An experimental demonstration of exploitation competition in an ongoing invasion. Ecology **77****,** 118-132. (10.2307/2265661)

[53] Raczyński M, Stoks R, Johansson F, Sniegula S. 2021 Size-mediated priority effects are trait-dependent and consistent across latitudes in a damselfly. Oikos **130**, 1535-1547. (10.1111/oik.08353)

[54] Raczyński M, Stoks R, Sniegula S. 2022 Warming and predation risk only weakly shape size-mediated priority effects in a cannibalistic damselfly. Sci. Rep. **12**, 17324. (10.1038/s41598-022-22110-6) 36243749 PMC9569353

[55] Ji J, Zhang Y-X, Saito Y, Takada T, Tsuji N. 2016 Competitive and predacious interactions among three phytoseiid species under experimental conditions (Acari: Phytoseiidae). Environ. Entomol. **45**, 46-52. (10.1093/ee/nvv162) 26496951

[56] Suutari E, Suhonen J, Rantala MJ, Salmela J. 2004 Intraguild predation and interference competition on the endangered dragonfly *Aeshna viridis*. Oecologia **140**, 135-139. (10.1007/s00442-004-1559-6) 15098120

[57] Palomar G, Wos G, Stoks R, Sniegula S. 2023 Latitude-specific urbanization effects on life history traits in the damselfly *Ischnura elegans*. Evol. Appl. **16**, 1503-1515. (10.1111/eva.13583) 37622092 PMC10445092

[58] Parmesan C, Yohe G. 2003 A globally coherent fingerprint of climate change impacts across natural systems. Nature **421**, 37-42. (10.1038/nature01286) 12511946

[59] Hickling R, Roy DB, Hill JK, Fox R, Thomas CD. 2006 The distributions of a wide range of taxonomic groups are expanding polewards. Global Change Biol. **12**, 450-455. (10.1111/j.1365-2486.2006.01116.x)

[60] Nagano K, Hiraiwa MK, Ishiwaka N, Seko Y, Hashimoto K, Uchida T, Sánchez-Bayo F, Hayasaka D. 2023 Global warming intensifies the interference competition by a poleward-expanding invader on a native dragonfly species. Figshare. (10.6084/m9.figshare.c.6927427) PMC10663793 38026017

